# Case Report: Concurrent neurofibromatosis type 1 with papillary thyroid carcinoma and gastrointestinal stromal tumor

**DOI:** 10.3389/fonc.2025.1529765

**Published:** 2025-07-10

**Authors:** Ruba Dweik, Jana Faroun, Rita Yacoub, Mohammad I. Smerat, Yousef Abu Asbeh

**Affiliations:** ^1^ Medical Research Club, Faculty of Medicine, Al-Quds University, Jerusalem, Palestine; ^2^ Department of Radiology, Al-Ahli Hospital, West Bank, Hebron, Palestine; ^3^ Department of Thoracic Surgery, Al-Ahli Hospital, Hebron, Palestine; ^4^ Faculty of Medicine, Al-Quds University, Jerusalem, Palestine

**Keywords:** neurofibromatosis type 1, papillary thyroid carcinoma, gastrointestinal stromal tumor, genetic counseling, case report

## Abstract

Neurofibromatosis type 1 (NF1) is a genetic disorder characterized by benign tumors such as neurofibromas and café-au-lait spots, with affected individuals at increased risk for malignant tumors, including gastrointestinal stromal tumors (GIST) and, rarely, papillary thyroid carcinoma (PTC). This case report presents a 30-year-old Palestinian woman with NF1 who experienced severe abdominal pain and melena, leading to the diagnosis of a jejunal GIST, which was surgically removed. Postoperative imaging revealed cervical and thoracic lesions. A follow-up PET scan indicated hypermetabolic masses in the thyroid and chest. Subsequent surgery confirmed the diagnosis of PTC and neurofibromas, with whole exome sequencing identifying a likely pathogenic variant in the NF1 gene. This case demonstrates the value of comprehensive evaluation and genetic counseling for NF1 patients due to the risk of multiple tumors, which points to careful monitoring for early detection and management. To our knowledge, this instance is the first reported case of concurrent GIST and PTC in a patient with NF1.

## Introduction

Neurofibromatosis type 1 (NF1), also known as von Recklinghausen disease, is a common autosomal dominant condition caused by mutations of the NF1 gene, located on chromosome 17q11.2 (1). The NF1 gene makes a protein called neurofibromin, which helps prevent tumors by turning off a process that can lead to cell growth, specifically by changing active RAS-GTP into inactive RAS-GDP, which helps control the RAS/MAPK pathway. Neurofibromin is found in high concentrations in melanocytes, Schwann cells, oligodendrocytes, and neurons. It influences how cells develop, how their structure changes, and how they die by working with different signaling pathways, such as PI3K/AKT/mTOR, cAMP/PKA, and Rho/ROCK (2). Loss of neurofibromin function due to defective mRNA or protein expression results in constitutive activation of RAS, leading to uncontrolled cell growth and cancer. Somatic mutations also drive sporadic cancers, including gliomas, neurofibromas, and malignant peripheral nerve sheath tumors (MPNST). Moreover, it can disrupt the mRNA transcript through splicing errors, premature stop codons, or altered coding sequences, leading to loss of functional neurofibromin. The NF1 gene has one of the highest rates of spontaneous mutation in humans; approximately 5,000 pathogenic variations, including large deletions and frameshift, nonsense, missense, and splicing mutations. Both germline and somatic mutations disrupt neurofibromin’s RAS/MAPK regulation, promoting proliferation in NF1-associated tumors (e.g., MPNSTs) and sporadic malignancies (3).

NF1 affects approximately 1 in 2,500 to 3,000 people, making it one of the most prevalent hereditary disorders (4). Clinical manifestations vary significantly among patients due to phenotypic heterogeneity. Despite the frequent identification of NF1 in early childhood, some individuals may not receive a diagnosis until their late 20s, 30s, or even later (5).

The hallmark of NF1 is the development of neurofibromas. They are generally benign and asymptomatic but can cause pain and damage to the surrounding tissues. They start in the cells and tissues of the nervous system and are the defining feature. Additional clinical features include pigmentation changes such as café-au-lait spots on the skin, as well as benign skin tumors or freckles. Brain tumors, anomalies of the bones, and other complications are examples of other clinical features the patients could develop (6).

Therapeutic interventions are determined according to the symptoms that appear on the patient; for example, mitogen-activated protein kinase (MEK) inhibitors (selumetinib, mirdametinib) for plexiform neurofibromas. For malignant peripheral nerve sheath tumors (MPNSTs), complete surgical resection is the only curative treatment for localized MPNST, while radiotherapy is considered for high-grade or incompletely resected tumors, and neoadjuvant chemotherapy with anthracycline and ifosfamide is the standard regimen for high-risk cases despite limited randomized data (7). Management involves annual dermatologic/neurologic exams, blood pressure monitoring, and whole-body MRI for high-risk patients, with women starting breast MRI at 30 due to elevated risk. Emerging therapies include NF1 mRNA replacement, RAS inhibitors, and anti-Transforming Growth Factor-beta (anti-TGF-β) for MPNSTs (8).

Optic nerve gliomas, glioblastomas, paragangliomas, malignant ganglioneuromas of peripheral nerves, breast cancer, leukemia, and rhabdomyosarcoma are among the neoplasms whose NF1 has been linked to an elevated risk (9). One of the most prevalent abdominal cancers associated with NF1 is gastrointestinal stromal tumors (GISTs) (10). Recent case reports have highlighted a connection between gastrointestinal stromal tumors (GISTs) and neurofibromatosis type 1 (NF1), but papillary thyroid carcinoma (PTC) remains rare among NF1 patients, with only three documented cases of large thyroid tumors in the literature.

Here, we present the first reported case of an unusual association of papillary thyroid carcinoma and gastrointestinal stromal tumor in a 30-year-old female patient who was previously diagnosed with NF1.

## Case presentation

A 30-year-old Palestinian female patient, diagnosed with NF1 at the age of 10 years, was in her usual state of health until November 2023, when she presented to our center complaining of severe abdominal pain for 2 days. The pain was colicky in nature, localized in the epigastric region and radiating to the mid-gastric region, and was associated with nausea and black tarry stool, with no fresh hematochezia or hematemesis.

Her personal background demonstrates that her parents are first-degree cousins, her father passed away from multiple myeloma, she has three healthy siblings, and none of her family members have a similar condition.

Physical examination showed that the patient was conscious, drowsy, and pale; multiple café au lait spots (more than 6 lesions measured >15 mm) with multiple skin lipomas were noticed all over her body. Abdominal examination showed mild left lower quadrant tenderness with palpable right upper quadrant cystic lesions, while neck examination was normal with no visible or palpable neck masses or lymph nodes; the rest of the physical examination was unremarkable.

**Figure 1:**
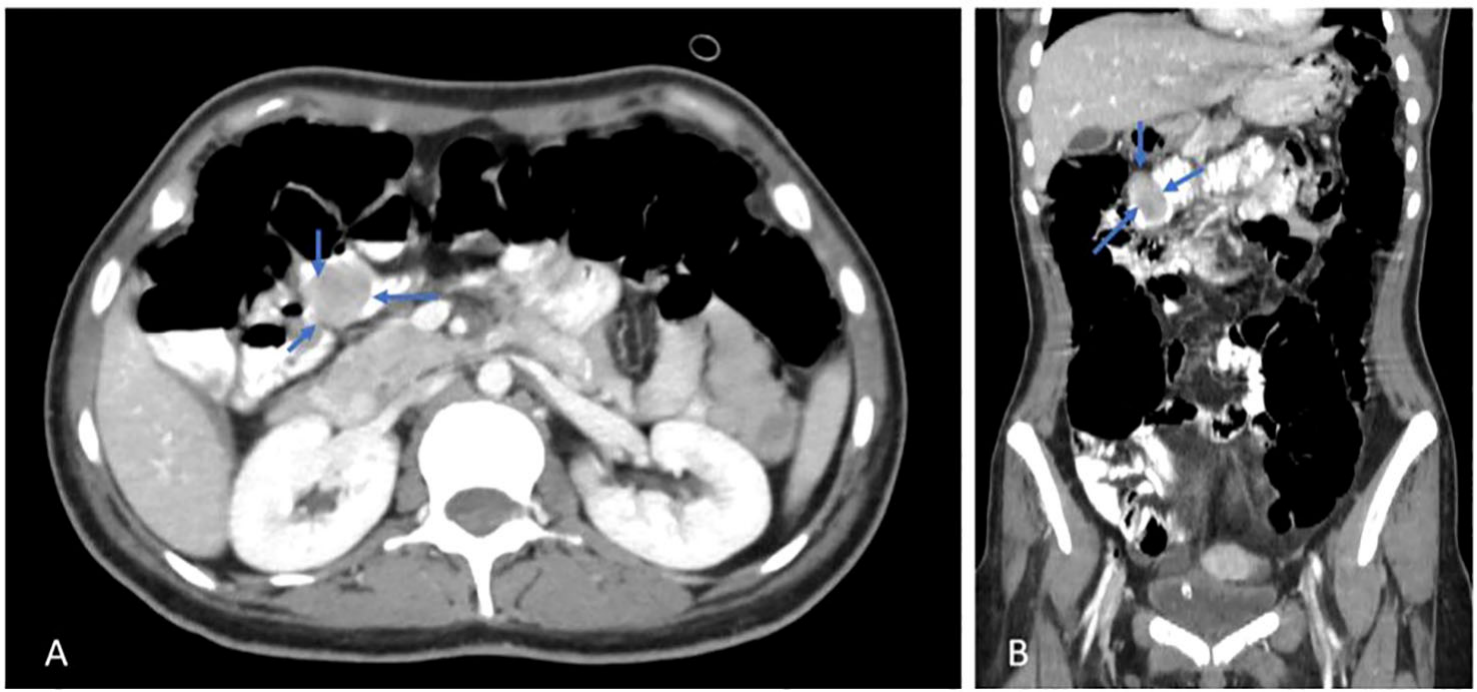
Axial **(A)** & Coronal **(B)** views, post contrast CT scan, shows a jejunal mass (blue arrows).

The gastrointestinal medical team was consulted, and further evaluation was recommended. For this reason, she underwent two sessions of upper gastroscopy that showed no active bleeding or other abnormalities. Then the patient underwent colonoscopy, and the colon and terminal ileum were full of altered blood. Pushed endoscopy was done, showing about 3–4 cm of rounded submucosal lesion with an ulcer at the tip and minimal bleeding at the proximal jejunum, suggesting GIST. Subsequently, abdominal computed tomography (CT scan) was ordered, which showed a well-defined intraluminal heterogeneous enhancing soft tissue lesion noted within the jejunal loop at the right upper abdomen, measuring about 2.5 x 2 cm, as shown in **Figure 1**.

Surgical resection and anastomosis were done for the jejunal mass. The mass was sent to the pathology lab, which confirmed the diagnosis of small bowel GIST in the jejunum. After the operation, the patient was sent back to the Intensive Care Unit (ICU), where she stayed for three days, and then was transferred to the surgical ward for another seven days.

The patient received two units of packed red blood cells (PRBCs) on admission due to a drop in hemoglobin from 8.9 to 5.5, and an additional five units over the next four days. She was also given six units of fresh frozen plasma (FFP) and two units of platelets before her operation.

**Figure 2:**
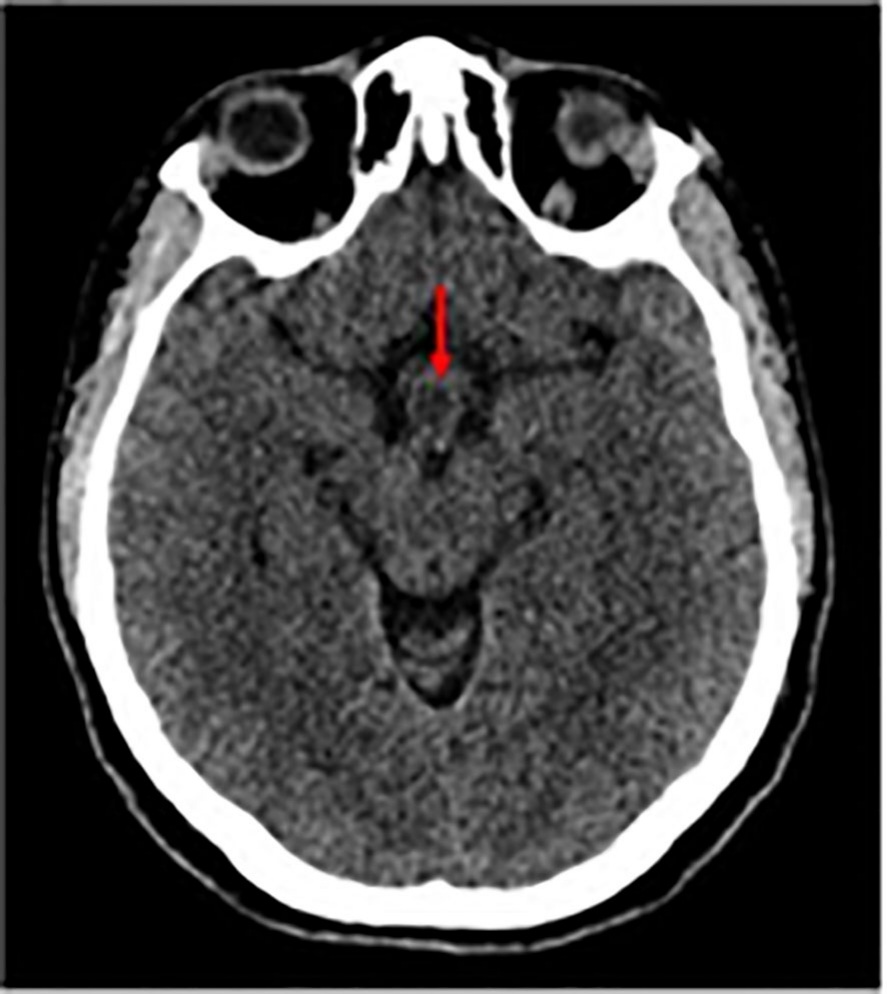
Non-enhanced Axial CT scan shows a hypodense lesion (red arrows).

A whole-body CT scan with intravenous contrast was performed during her hospital stay, and the results were as follows: Brain CT Scan: A suspected small hypodense lesion was seen in the hypothalamic area measuring about 1 cm in diameter as shown in **Figure 2**.

Non-enhanced Axial CT scan shows a hypodense lesion (red arrows).

### Neck and chest CT scan

**Figure 3:**
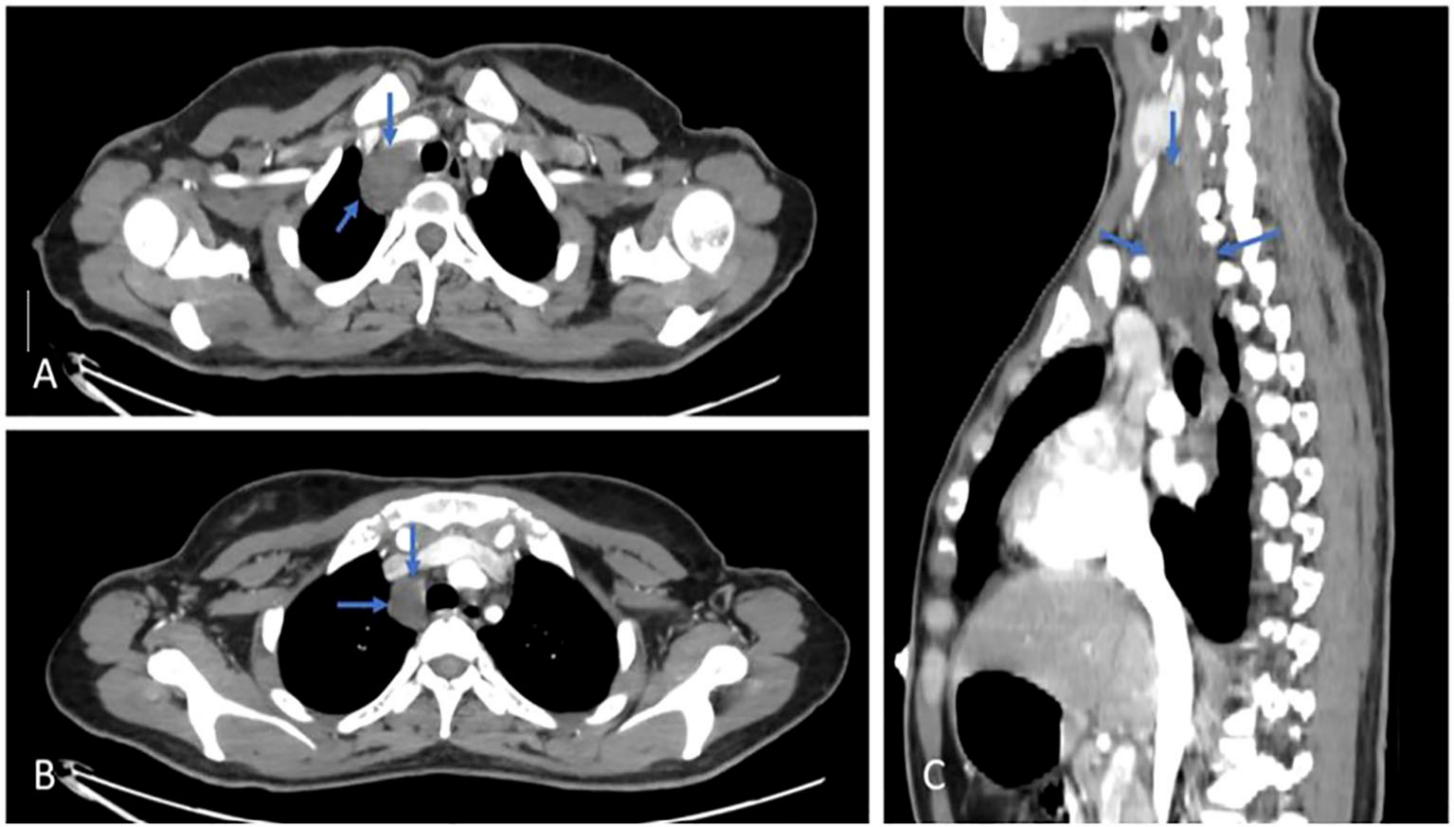
Axial **(A, B)** & Sagittal **(C)** views, post contrast CT scan, shows a cervical lesion (blue arrows).

There was a large, well-defined lesion at the right lower cervical region (carotid space) extending inferiorly into the superior mediastinum along the right tracheal wall, measuring about 5 x 3 cm, mostly suggestive of neurofibroma, as shown in **Figure 3**.

**Figure 4:**
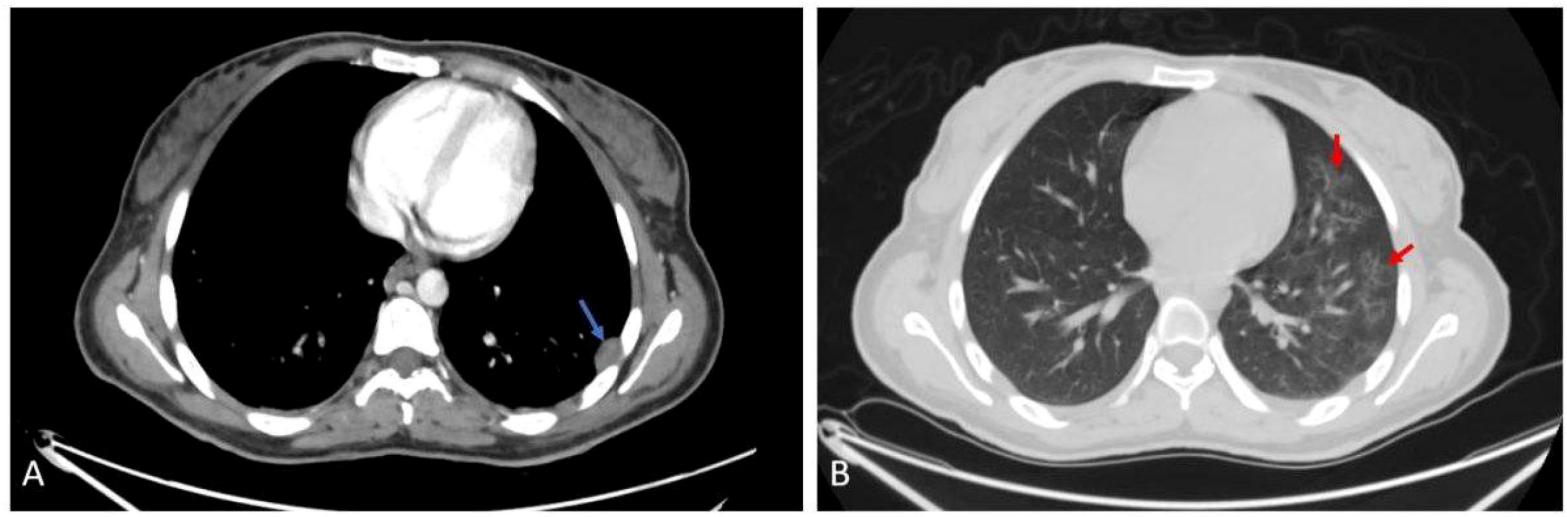
Axial **(A)** post contrast CT scan shows left pleurally based nodule (blue arrow). Axial **(B)** CT scan shows nodular and ground glass infiltration (red arrow).

The right thyroid gland shows a well-defined lesion measuring approximately 1.5 cm. There was also a pleural-based enhancing soft tissue nodule in the lateral segment of the left lower lung lobe, with diffuse nodular and branching ground-glass opacities, measuring about 20 x 13 mm. Another hypodense fusiform intramuscular lesion showing tapered ends and central enhancement was noted at the right lateral chest wall near the margin of the right 10th costal cartilage, as shown in **Figure 4**.

### Abdomen CT scan

There was a well-defined intraluminal heterogeneous enhancing soft tissue lesion noted within the jejunal loop at the right upper abdomen, measuring about 2.5 x 2 cm, and a bilateral polycystic ovarian morphology was noted with mild pelvic free fluid. The rest of the scan was normal, as shown in **Figure 1**.

The patient was discharged in good condition and advised to follow up with oncology, undergo genetic testing, and have a positron emission tomography (PET) scan.

Two months later, the patient presented again complaining of painful right lower chest swelling (about 2.5*2.5 rounded, firm, and mobile mass). The pain was intermittent, increasing in intensity over time and exacerbating when lying on the right side. Then she was admitted for subcutaneous chest mass excision. The excision was done with an uneventful postoperative course, and the pathological evaluation for the mass matched with neurofibromatosis.

**Figure 5:**
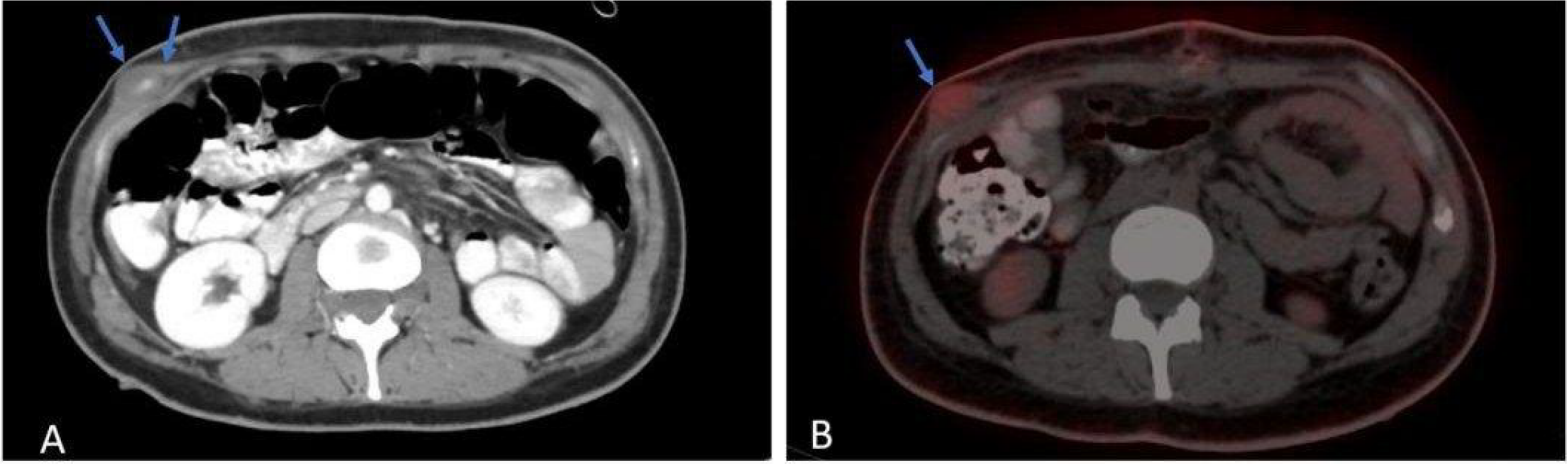
Axial **(A)** post contrast CT scan shows intramuscular lesion, with central enhancement (blue arrows). Axial **(B)** PET-CT scan shows hypermetabolic activity.

During her admission, a PET scan was done and showed multiple chest nodules and a hypermetabolic right upper abdominal lower chest mass (2 x 1 cm) with fludeoxyglucose F18 (FDG) uptake, as shown in **Figure 5**.

**Figure 6:**
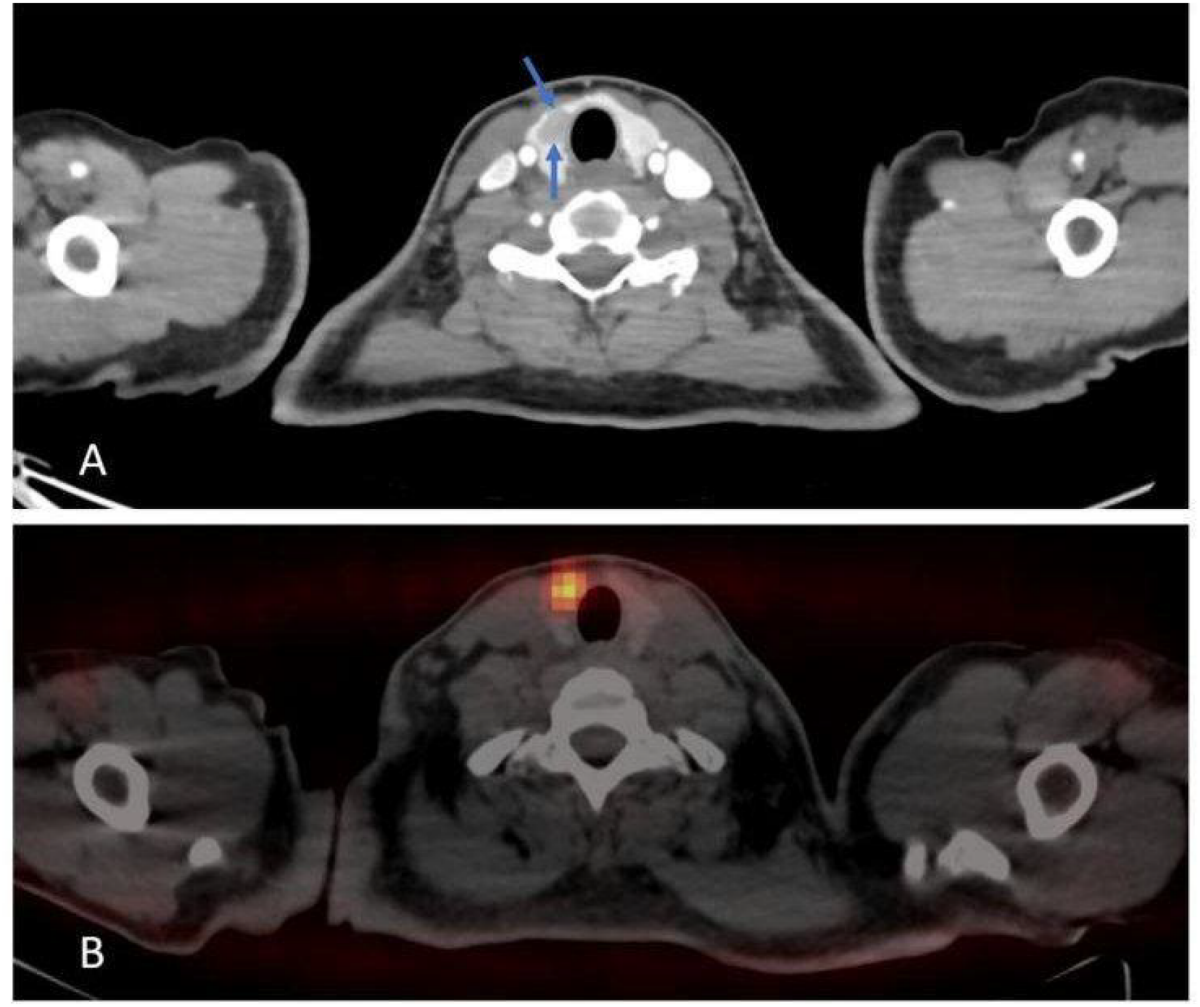
Axial **(A)** post contrast CT scan shows right thyroid nodule. Axial **(B)** PET-CT scan shows hypermetabolic activity.

Right thyroid nodule (1.1 cm) with hypermetabolic uptake and right lower cervical paratracheal soft tissue (3.5 x 2.6 cm). as shown in **Figure 6**.

Clinical evaluation and all needed labs were performed; thyroid stimulating hormone level (TSH) was normal and measured 1.9 uIU (normal range: 0.4–4.94 uIU), and the other labs were within the normal range.

As per the oncologist’s recommendation, she had an ultrasound-guided biopsy (US) and fine needle aspiration (FNA) for the right thyroid nodule. The biopsy showed papillary thyroid carcinoma (PTC). Bethesda guidelines category VI. She underwent total thyroidectomy and right-sided neck dissection with an uneventful postoperative course. The pathological report showed cellular smears composed of sheets, papillae, and microfollicles. The cells show nuclear changes of papillary thyroid carcinoma in the form of grooves, pseudoinclusions, membrane thickening, nuclear crowding, powdery chromatin, and variable cytoplasm (scant, squamoid, Hurthle-like, and vacuolated). Histiocytes, including multinucleated giant cells, were also detected.

Our patient then was referred to a genetic clinic and recommended to do a germline whole exome sequencing test, which emphasized the presence of a heterozygous likely pathogenic variant in the NF1 gene (c.2793-2795delAAT (p.Met932del)). The genetic finding was consistent with the suspected clinical diagnosis.

**Table 1** demonstrates the timeline of the patient’s presentation.

**Table 1:** Timeline of the patient’s clinical presentation, diagnostic investigations, treatment procedures, and follow-up events.

Date	Description
23/11/2023	The patient came to the ER complaining of a black tarry stool for 2 days.
23/11/2023	Gastroscopy procedure.
25/11/2023	Colonoscopy procedure.
26/11/2023	Capsule endoscopy procedure.
27/11/2023	Gastroscopy procedure.
28/11/2023	Whole body CT scan
29/11/2023	Small bowel resection and removal of GIST tumor.
30/11/2023	The pathology report indicated the presence of a GIST spindle cell type.
28/12/2023	The PET results.
23/2/2024	The patient has been complaining of swelling in the right lower chest for one year.
3/3/2024	Outpatient visit, which revealed that the patient have chest wall mass and the us guided biopsy showed papillary thyroid carcinoma
18/4/2024	Patient came complaining of incidental finding of thyroid nodule
19/4/2024	Patient underwent total thyroidectomy and neck dissection
6/5/2024	Initial report for having a positive result on whole exome sequencing, including the central nervous system and mitochondrial genome.
1/6/2024	Genetic consultation

## Discussion

Neurofibromatosis type 1 is a multisystem disorder that affects 1 in 3,000s (11). The primary cause of NF1 is a mutation of the NF1 gene on chromosome 17q11. 2 (1). Numerous clinical traits that might manifest at any age are linked to NF1. Every racial and ethnic group is affected, and both sexes are equally affected (12). Neurofibromatosis type 1 is characterized by several café-au-lait macules (CALMs), cutaneous neurofibromas (CNs) (12), plexiform neurofibromas, deep or subcutaneous nodular neurofibromas, and distinctive ocular symptoms. Individuals with NF1 are more likely to experience difficulties with behavior, learning, and social adaptation. Optic or non-optic brain tumors occur far more frequently; however, the majority of these tumors have a benign course. People with NF1, particularly those with large numbers or big-size plexiform or deep nodular neurofibromas, are more likely to develop malignant peripheral nerve sheath tumors that tend to emerge at a much younger age and have a poorer prognosis than in the general population (13).

NF1 can be diagnosed by applying the diagnostic criteria established by the National Institutes of Health (NIH). To make the diagnosis of NF1, an individual must meet a minimum of two of the following requirements: (a) possessing six or more café-au-lait spots measuring more than 5mm in the prepubertal period and more than 15 mm in the post-pubertal period, (b) a growing plexiform neurofibroma or several other neurofibromas, (c) freckling in the groin area or armpits, (d) having an optic glioma, (e) at least two growing Lisch nodules (hamartomas), (f) displaying specific skeletal abnormalities, or (g) having a first-degree relative with NF1 (14). Based on the initial two diagnostic criteria, our patient was identified as having NF1. The patient developed GIST after being diagnosed with NF1. A period of time later, the patient was then diagnosed with PTC.

It is well established that NF1 predisposes to cancers (15). Patients with NF1 have an increased risk of various tumors. The most common are non-neurofibroma neoplasms (28.1%), followed by low-grade gliomas (22.3%), particularly optic pathway gliomas (16.5%). Sarcomas, such as malignant peripheral nerve sheath tumors (4.5%) and breast cancer (1.9%), and Gastrointestinal stromal tumors (0.6%) also occur. Less frequent tumors include endocrine neoplasms (pheochromocytoma, neuroendocrine tumors), skin cancers (basal cell carcinoma), leukemias, genitourinary cancers, lymphomas, and rare cases of lung cancer, meningioma, and others. This diverse tumor spectrum underscores the need for vigilant monitoring in NF1 patients (16).

GIST is one of the most prevalent non-neurological malignancies compared to the general population; NF1 individuals had a 45 times greater incidence of GIST (17). Which is a mesenchymal tumor typically originating in the stomach or small intestine (18). In contrast to sporadic GIST, NF1-associated GIST is more frequent in women and manifests at a younger age (19). Our patient has one tumor in the jejunum. Our patient’s gastroscopy showed no abnormalities or active bleeding. Therefore, abdominal CT or magnetic resonance imaging should be performed on middle-aged and elderly NF1 patients who have gastrointestinal symptoms to rule out GIST in the intestines. A CT scan of the abdomen can reveal tumors and provide details about their size, location, edges, and how they interact with nearby tissues. Capsule endoscopy and small intestinal endoscopy are also commonly used to diagnose GISTs (19). The diagnosis for our patient was confirmed by conducting an abdominal CT scan and capsule endoscopy. On the other hand, the case took an unexpected turn when a thyroid nodule with hypermetabolic uptake was subsequently discovered on a PET scan. Additional tests, which included ultrasound-guided biopsy and FNA, revealed PTC, demonstrating the complex nature of the patient’s medical condition. Endocrine tumors, like pheochromocytomas, are more common in people with NF1, with a percentage of 0.1% to 5.7% of NF1 individuals (20), but PTC appears to be quite uncommon in NF1 patients (21). The literature currently has very few instances demonstrating NF1 with PTC, but none of them showed the co-existence of GIST and PTC with NF1.

No family history of NF1 was present in the patient, except for her father, who passed away from multiple myeloma (MM). The association between MM and NF1 is very rarely described in the literature. The data did not support a direct link between NF1 and MM pathogenesis (22). This study has potential limitations; one of the patient’s case limitations was the lack of a childhood neurofibromatosis diagnosis. Furthermore, the diagnosis procedure would have been delayed, and significant expenditures associated with genetic testing.

To the best of our knowledge, this case is the first case combining GIST, PTC, and NF1 in one patient, and that’s what makes our case report unique.

The fact that GIST occurs together with PTC in this NF1 case draws attention to the intimate connection between genetics and the formation of tumors. Physicians should always bear in mind that there are many forms of cancer in people suffering from NF1, like in this patient’s case. The point also emphasizes the care taken during genetic testing when making treatment strategies, indicating a probable relationship between genes and the development of tumors among patients suffering from NF1. Research is needed to understand the complex mechanisms behind the different manifestations and tumors that occur in individuals with NF1.

## Data Availability

The original contributions presented in the study are included in the article/supplementary material. Further inquiries can be directed to the corresponding author.
